# Impact of Silymarin Supplementation on Liver Function and Enzyme Profiles in Diverse Chronic Liver Disease Etiologies

**DOI:** 10.7759/cureus.76313

**Published:** 2024-12-24

**Authors:** Muhammad Zohaib Amjad, Muhammad Umair Hassan, Mahnoor Rehman, Arslan Shamim, Hafiz Muhammad Zeeshan, Zohaib Iftikhar, Adeel Ahmed, Muhammad Irfan Jamil

**Affiliations:** 1 Internal Medicine, Royal Berkshire Hospital, Reading, GBR; 2 Internal Medicine, Punjab Medical College, Faisalabad, PAK; 3 Gastroenterology, Lahore General Hospital, Lahore, PAK; 4 Internal Medicine, Lahore General Hospital, Lahore, PAK; 5 Internal Medicine, Nishtar Medical University, Multan, PAK; 6 Medicine, Lahore General Hospital, Lahore, PAK; 7 Nephrology, Lahore General Hospital, Lahore, PAK

**Keywords:** child-turcotte-pugh (ctp) score, chronic liver disease (cld), hepatitis b virus (hbv), nonalcoholic fatty liver disease (nafld), silymarin

## Abstract

Aim and background: This study aimed to evaluate the efficacy of silymarin in improving liver function and reducing liver stiffness in chronic liver disease (CLD) patients. Silymarin, a hepatoprotective agent, has shown potential benefits in non-alcoholic fatty liver disease (NAFLD) and liver fibrosis, but evidence in CLD with varied etiologies remains limited. This study addresses the gap by assessing its impact across diverse etiological subgroups.

Material and method: A non-randomized clinical trial was conducted at Lahore General Hospital, Lahore, over 18 months. Out of 148 enrolled chronic liver disease (CLD) patients, 141 completed the 12-week follow-up. Patients were stratified into two groups: silymarin (200 mg twice daily) plus standard care and standard care alone. Baseline and follow-up data, including clinical, biochemical, and FibroScan (EchoSens, Ivry-sur-Seine, France) was collected and stratified analysis based on etiology was performed using Statistical Package for the Social Sciences (SPSS) version 26 (IBM Corp., Armonk, NY).

Results: This study evaluated 141 chronic liver disease (CLD) patients who completed the three-month follow-up, 68 in the standard treatment group and 73 in the silymarin group. Baseline characteristics were comparable except for age and body mass index (BMI). Alanine aminotransferase (ALT) levels significantly reduced in the silymarin group (63.04 ± 22.38 U/L) compared to the standard group (78.49 ± 22.93 U/L, p=0.000), with higher ALT normalization in the silymarin group (35.6% vs. 22.1%, p=0.076). Aspartate aminotransferase (AST) levels were also significantly lower in the silymarin group (57.08 ± 20.94 U/L vs. 65.90 ± 24.18 U/L, p=0.022). Improvements in hepatic encephalopathy, ascites, and Child-Turcotte-Pugh (CTP) class were similar across groups (p > 0.05). The stratified analysis highlighted greater ALT and AST reductions for hepatitis B virus (HBV) and non-alcoholic fatty liver disease (NAFLD) in the silymarin group. FibroScan (EchoSens, France) scores, bilirubin, albumin, international normalized ratio (INR), and prothrombin time (PT) showed no significant differences between groups. These findings suggest silymarin’s potential in improving liver enzymes, particularly in metabolic etiologies.

Conclusion: This study demonstrates that silymarin effectively reduces ALT and AST levels and achieves higher ALT normalization compared to standard treatment in CLD patients. While improvements in hepatic encephalopathy, ascites, and CTP class were comparable between groups, silymarin showed greater efficacy in metabolic conditions like NAFLD.

## Introduction

Chronic liver disease (CLD) encompasses a spectrum of disorders, including non-alcoholic fatty liver disease (NAFLD), hepatitis C virus (HCV)-induced cirrhosis, and alcohol-induced liver damage, affecting millions globally. The prevalence of CLD is on the rise, attributed to factors such as HCV, hepatitis B virus (HBV), obesity, diabetes, and excessive alcohol consumption [1,2]. Supportive treatments for CLD vary, ranging from lifestyle modifications to pharmacological interventions like metformin, pioglitazone, and vitamin E [3,4]. Among these, silymarin, a natural extract from the milk thistle plant, has garnered attention for its potential hepatoprotective effects. However, despite its widespread use, the recent literature challenges in defining the precise role of silymarin in managing CLD, particularly in terms of its impact on mortality, quality of life, and specific liver function metrics beyond basic biochemical improvements [5,6].

The current state of knowledge on silymarin's role in treating CLD presents a landscape marked by both hopeful outcomes and areas of disagreement. Li et al. (2024) provided a comprehensive meta-analysis demonstrating silymarin's effectiveness in improving lipid profiles, insulin resistance, and liver injury markers in patients with non-alcoholic fatty liver disease (NAFLD), highlighting its potential for biochemical modulation in liver disease [7]. Similarly, Chahkandi et al. (2023) reported significant biochemical improvements in CLD patients treated with silymarin, specifically in liver enzymes [8]. Studies by Hashemi et al. (2009) and Wah Kheong et al. (2017) further underscored silymarin's efficacy in reducing serum levels of alanine aminotransferase (ALT) and aspartate aminotransferase (AST), and in achieving fibrosis reduction based on histology and liver stiffness measurements, suggesting its role in fibrosis management in non-alcoholic steatohepatitis (NASH) patients [9,10]. Conversely, another revealed no significant histological improvement in NASH compared to placebo [5]. Fathalah et al. (2017) demonstrating significant benefits of high-dose silymarin in chronic HCV-decompensated cirrhotic patients, contribute to the existing debate [11].

Among the available treatments, silymarin emerges as a potential option. However, existing studies present are not consistent regarding its efficacy and secondly, most of the available literature that favors its use predominantly focuses on non-alcoholic fatty liver disease (NAFLD), leaving a gap in understanding its effects on CLD arising from other etiologies. This study aimed to fill the gap by examining the silymarin's role in different etiologies of CLD. It explored silymarin's effect on liver enzymes, symptomatology, fibrosis, and Child-Turcotte-Pugh (CTP) class in patients with CLD, which is one of the most commonly encountered diseases.

## Materials and methods

It was a non-randomized clinical trial conducted at the Department of Gastroenterology, Lahore General Hospital, Lahore after obtaining ethical approval from the institutional review board (Approval No.: PGMI/AMC/LGH/2021/09/133), over a duration of 18 months from February 2023 to August 2024. After explaining the purpose, risks, and benefits of the study, informed consent was taken from 148 patients. The sample size was calculated through the two-proportion formula by estimating the frequency of improved liver stiffness measured by FibroScan (EchoSens, Ivry-sur-Seine, France) in the silymarin group as 24.2% and in the placebo group as 2.3% [10]. Patients aged 20-70 years diagnosed with CLD regardless of the etiology, through clinical stigmata (ascites, spider angiomas, palmar erythema, etc.) and radiologically (shrunken liver, altered echotexture, irregular margins, splenomegaly, portal vein >12 mm, or FibroScan ≥7.1 kPa) and classified as Child-Turcotte-Pugh A or B were included in the study. Patients with acute liver failure, serious comorbidities (e.g., ESRD {end-stage renal disease}, uncontrolled diabetes, heart failure), candidate for a liver transplant, active malignancies, recently used hepatoprotective drug use (vitamin E, pioglitazone, or metformin), using antivirals and silymarin hypersensitivity were excluded. Data related to demographic, clinical, and laboratory details including age, gender, BMI, duration of liver disease, etiology, clinical symptoms, liver function tests, and FibroScan (EchoSens, France) was collected. Following baseline assessments, participants were divided into two groups. Group A received Tab silymarin 200 mg twice daily along with standard care. Group B received standard care only. Standard care included dietary counseling, anti-encephalopathy measures, portal hypertension management, and avoiding hepatotoxic drugs.

All participants enrolled in the study, irrespective of their assignment to the silymarin or placebo groups, will receive the standard treatment regimen for CLD as per the current clinical guidelines and best practices. This comprehensive care approach includes dietary recommendations, such as reduced sodium intake for those with ascites, and the management of complications associated with CLD like variceal bleeding, encephalopathy, and infection control. Pharmacological interventions will be tailored to individual patient needs, encompassing medications such as diuretics for ascites management, beta-blockers for variceal bleeding prophylaxis, and lactulose or rifaximin for hepatic encephalopathy. Follow-up at three months included symptoms, laboratory, and liver stiffness assessment, Data was collected using data collection proforma.

The Statistical Package for the Social Sciences (SPSS), version 26 (IBM Corp., Armonk, NY) was used for data analysis. Continuous variables such as liver enzymes, FibroScan (EchoSens, France) score, and CTP class score were presented as means and standard deviation. Categorical variables like symptoms and CTP class were summarized as frequencies and percentages. The Chi-square test was used for the comparison of categorical and continuous variables were analyzed using the student’s t-test. Potential confounding variables were stratified, and after stratification, their effects on the primary outcome were assessed. A p-value of < 0.05 was considered statistically significant.

## Results

Out of the initial 148 participants, 141 completed the study; 68 (48.2%) in the standard treatment group and 73 (51.8%) in the silymarin group. The majority were male participants (97, 68.8%), and 44 (31.2%) were female participants. The mean age of participants was 51.7 ± 8.33 years, with a mean CLD duration of 2.9 ± 1.51 years. The most common CLD etiology was HCV (76, 53.9%), followed by HBV (33, 23.4%), NAFLD (26, 18.4%), and alcoholic liver disease (6, 4.3%).

Baseline characteristics were equally distributed between groups in demographics, etiology, or liver parameters, except for age and BMI (Table 1).

**Table 1:** Comparison of baseline characteristics of patients in the standard treatment and silymarin groups. The table presents the baseline characteristics of patients in the standard treatment and silymarin groups. Categorical variables (e.g., gender, CTP class, etiology, ascites, and encephalopathy) were analyzed using the Chi-square test, while continuous variables (e.g., age, BMI, FibroScan score, and liver function tests) were compared using the independent t-test. A p-value <0.05 was considered statistically significant. CTP: Child-Turcotte-Pugh, CLD: chronic liver disease, HCV: hepatitis C virus, HBV: hepatitis B virus, NAFLD: non-alcoholic fatty liver disease, ALT: alanine aminotransferase, AST: aspartate aminotransferase, BMI: Body Mass Index, INR: International Normalized Ratio, PT: prothrombin time. Normal ranges: ALT/AST: 7-56 U/L, bilirubin: 0.1-1.2 mg/dL, albumin: 3.5-5.0 g/dL, INR: 0.8-1.2, PT: 10-13 seconds.

Baseline characteristics		Standard treatment group (n=68)	Silymarin group (n=73)	p-value
Gender	Male	50 (73.5%)	47 (64.4%)	0.242
	Female	18 (26.5%)	26 (35.6%)	
CTP class baseline	Class A	33 (48.5%)	40 (54.8%)	0.457
	Class B	35 (51.5%)	33 (45.2%)	
Etiology of CLD	HCV	33 (48.5%)	43 (58.9%)	0.427
	HBV	20 (29.4%)	13 (17.8%)	
	NAFLD	12 (17.6%)	14 (19.2%)	
	Alcoholic liver disease	3 (4.4%)	3 (4.1%)	
Presence of ascites	None	39 (57.4%)	43 (58.9%)	0.901
	Mild	24 (35.3%)	26 (35.6%)	
	Moderate	5 (7.4%)	4 (5.5%)	
Hepatic encephalopathy	None	56 (82.4%)	61 (83.6%)	0.849
	Grade 1-2	12 (17.6%)	12 (16.4%)	
Age (years)	-	53.25 ± 7.64	50.26 ± 8.73	0.033
Duration of CLD (years)	-	3.06 ± 1.41	2.75 ± 1.59	0.231
BMI (kg/m²)	-	26.89 ± 2.79	27.93 ± 3.04	0.037
FibroScan score (kPa)	-	11.32 ± 1.20	11.04 ± 1.13	0.148
ALT (U/L)	-	96.50 ± 16.25	94.18 ± 17.33	0.414
AST (U/L)	-	89.88 ± 23.10	86.52 ± 21.31	0.370
Bilirubin (mg/dL)	-	1.78 ± 0.39	1.77 ± 0.33	0.915
Albumin (g/dL)	-	3.59 ± 0.47	3.57 ± 0.46	0.816
INR	-	1.48 ± 0.41	1.46 ± 0.35	0.749
PT (seconds)	-	16.51 ± 3.82	16.34 ± 3.30	0.774

At three months, hepatic encephalopathy was absent in 89.7% of patients in the standard treatment group and 87.7% in the Silymarin group (p=0.703). CTP Class A was noted in 64.7% of the standard group and 67.1% of the silymarin group (p=0.762). Normalization of ALT occurred in 22.1% of the standard group and 35.6% of the silymarin group (p=0.076). Similarly, normalization of AST was observed in 30.9% of the standard group and 38.4% of the silymarin group (p=0.352). FibroScan (EchoSens, France) scores were similar between groups (9.58 ± 1.13 kPa in the standard group vs. 9.33 ± 1.04 kPa in the silymarin group, p=0.170). ALT levels were significantly lower in the silymarin group (63.04 ± 22.38 U/L) compared to the standard group (78.49 ± 22.93 U/L, p=0.000). AST levels were also significantly lower in the silymarin group (57.08 ± 20.94 U/L) compared to the standard group (65.90 ± 24.18 U/L, p=0.022). Other markers, including bilirubin, albumin, and PT showed no significant differences between groups (p-value > 0.05) (Table 2).

**Table 2:** Comparison of outcomes between standard treatment and silymarin groups at three months. This table compares the outcomes between standard treatment and silymarin groups at three months. Categorical variables (e.g., hepatic encephalopathy, ascites, CTP class, normalization of ALT/AST) were analyzed using the Chi-square test, while continuous variables (e.g., BMI, FibroScan score, liver function tests) were compared using the independent t-test. A p-value <0.05 was considered statistically significant. CTP: Child-Turcotte-Pugh, CLD: chronic liver disease, ALT: alanine aminotransferase, AST: aspartate aminotransferase, BMI: Body Mass Index, INR: International Normalized Ratio, PT: prothrombin time. Normal ranges: ALT/AST: 7-56 U/L, bilirubin: 0.1-1.2 mg/dL, albumin: 3.5-5.0 g/dL, INR: 0.8-1.2, PT: 10-13 seconds.

Outcomes		Standard treatment group (n=68)	Silymarin group (n=73)	p-value
Hepatic encephalopathy	None	61 (89.7%)	64 (87.7%)	0.703
	Grade 1-2	7 (10.3%)	9 (12.3%)	
Presence of ascites	None	46 (67.6%)	49 (67.1%)	0.845
	Mild	19 (27.9%)	22 (30.1%)	
	Moderate	3 (4.4%)	2 (2.7%)	
CTP class	Class A	44 (64.7%)	49 (67.1%)	0.762
	Class B	24 (35.3%)	24 (32.9%)	
Normalization of ALT	Yes	15 (22.1%)	26 (35.6%)	0.076
	No	53 (77.9%)	47 (64.4%)	
Normalization of AST	Yes	21 (30.9%)	28 (38.4%)	0.352
	No	47 (69.1%)	45 (61.6%)	
BMI (kg/m²)	-	26.69 ± 2.59	27.81 ± 2.66	0.012
FibroScan score (kPa)	-	9.58 ± 1.13	9.33 ± 1.04	0.170
CTP score	-	6.19 ± 1.16	6.01 ± 1.15	0.364
ALT (U/L)	-	78.49 ± 22.93	63.04 ± 22.38	0.000
AST (U/L)	-	65.90 ± 24.18	57.08 ± 20.94	0.022
Bilirubin (mg/dL)	-	1.58 ± 0.40	1.56 ± 0.33	0.755
Albumin (g/dL)	-	3.68 ± 0.43	3.69 ± 0.41	0.940
INR	-	1.27 ± 0.40	1.25 ± 0.35	0.746
PT (seconds)	-	14.51 ± 3.82	14.34 ± 3.30	0.774

Significant differences were observed in ALT normalization, which was higher in the silymarin group for HBV (23.1% vs. 10.0%, p=0.038), and ALT levels, with lower mean values in the silymarin group for HCV (67.58 ± 22.41 U/L vs. 80.91 ± 23.64 U/L, p=0.014) and NAFLD (42.43 ± 16.90 U/L vs. 67.92 ± 30.47 U/L, p=0.013). Similarly, AST levels were significantly lower in the silymarin group for HBV (54.46 ± 17.14 U/L vs. 72.50 ± 19.60 U/L, p=0.011) and NAFLD (42.86 ± 15.97 U/L vs. 60.58 ± 20.51 U/L, p=0.021). No significant differences were noted in FibroScan (EchoSens, France) scores across etiologies (e.g., HCV: 9.39 ± 0.96 vs. 9.48 ± 1.10, p=0.678; HBV: 9.28 ± 1.11 vs. 10.02 ± 1.25, p=0.094) (Table 3).

**Table 3:** Outcome stratified by etiologies and treatment groups. This table presents outcomes stratified by the etiology of chronic liver disease (HCV, HBV, NAFLD, and alcoholic liver disease) and treatment groups (standard vs. silymarin). Categorical outcomes, including normalization of ALT, AST, CTP class A, absence of hepatic encephalopathy, and absence of ascites, were analyzed using Chi-square or Fisher’s exact tests. Continuous variables, such as FibroScan score, ALT, AST, bilirubin, albumin, INR, and PT, were compared using independent t-tests. Significant differences were observed for ALT normalization (p=0.038 for HBV), ALT levels (p=0.014 for HCV and p=0.013 for NAFLD), and AST levels (p=0.011 for HBV and p=0.021 for NAFLD). Other outcomes, including FibroScan score, bilirubin, albumin, INR, PT, and the majority of categorical variables, were not statistically significant (p > 0.05), suggesting comparable results between the treatment groups for these parameters. CTP: Child-Turcotte-Pugh, CLD: chronic liver disease, HCV: hepatitis C virus, HBV: hepatitis B virus, NAFLD: non-alcoholic fatty liver disease, ALT: alanine aminotransferase, AST: aspartate aminotransferase, BMI: Body Mass Index, INR: International Normalized Ratio, PT: prothrombin time. Normal ranges: ALT/AST: 7-56 U/L, bilirubin: 0.1-1.2 mg/dL, albumin: 3.5-5.0 g/dL, INR: 0.8-1.2, PT: 10-13 seconds.

Outcome Variable	Etiologies of CLD							
	HCV		HBV		NAFLD		Alcoholic liver disease	
	Treatment groups							
	Standard	Silymarin	Standard	Silymarin	Standard	Silymarin	Standard	Silymarin
Normalization of ALT	6 (18.2%)	12 (27.9%)	2 (10.0%)	3 (23.1%)	6 (50.0%)	11 (78.6%)	1 (33.3%)	0 (0.0%)
Normalization of AST	16 (48.5%)	13 (30.2%)	2 (10.0%)	5 (38.5%)	3 (25.0%)	10 (71.4%)	0 (0.0%)	0 (0.0%)
CTP class A	21 (63.6%)	27 (62.8%)	13 (65.0%)	10 (76.9%)	7 (58.3%)	9 (64.3%)	3 (100.0%)	3 (100.0%)
No hepatic encephalopathy	29 (87.9%)	39 (90.7%)	18 (90.0%)	9 (69.2%)	11 (91.7%)	13 (92.9%)	3 (100.0%)	3 (100.0%)
No ascites	26 (78.8%)	29 (67.4%)	10 (50.0%)	9 (69.2%)	7 (58.3%)	8 (57.1%)	3 (100.0%)	3 (100.0%)
FibroScan score (Mean ± SD)	9.48 ± 1.10	9.39 ± 0.96	10.02 ± 1.25	9.28 ± 1.11	9.23 ± 0.90	9.36 ± 1.30	9.10 ± 0.87	8.50 ± 0.36
ALT (U/L, Mean ± SD)	80.91 ± 23.64	67.58 ± 22.41	81.05 ± 15.38	68.46 ± 17.58	67.92 ± 30.47	42.43 ± 16.90	77.00 ± 21.79	70.67 ± 8.62
AST (U/L, Mean ± SD)	58.12 ± 19.24	59.98 ± 19.90	72.50 ± 19.60	54.46 ± 17.14	60.58 ± 20.51	42.86 ± 15.97	128.67 ± 13.50	93.33 ± 20.82
Bilirubin (mg/dL, Mean ± SD)	1.64 ± 0.43	1.58 ± 0.30	1.54 ± 0.39	1.55 ± 0.33	1.56 ± 0.33	1.46 ± 0.41	1.30 ± 0.40	1.77 ± 0.31
Albumin (g/dL, Mean ± SD)	3.66 ± 0.46	3.65 ± 0.44	3.68 ± 0.45	3.71 ± 0.25	3.73 ± 0.37	3.71 ± 0.46	3.73 ± 0.38	4.07 ± 0.06
INR (Mean ± SD)	1.31 ± 0.44	1.26 ± 0.35	1.22 ± 0.35	1.17 ± 0.31	1.17 ± 0.39	1.25 ± 0.34	1.45 ± 0.37	1.32 ± 0.59
PT (seconds, Mean ± SD)	14.94 ± 4.18	14.49 ± 3.33	14.10 ± 3.34	13.62 ± 2.99	13.58 ± 3.75	14.43 ± 3.25	16.33 ± 3.51	15.00 ± 5.57

## Discussion

The global burden of CLD encompasses a diverse array of etiologies, including viral hepatitis, alcoholic liver disease, and NAFLD, presenting significant challenges in clinical management and patient quality of life. Among the therapeutic options explored, silymarin, extracted from the milk thistle plant (*Silybum marianum*), has been historically recognized for its hepatoprotective properties, offering a complementary approach to conventional treatments [6,12].

This study reported a significant reduction in ALT levels in the silymarin group after three months, with baseline levels decreasing from 94.18 ± 17.33 U/L to 63.04 ± 22.38 U/L (p = 0.000) in the silymarin group compared to standard care. This reduction aligns with findings from previous literature. Famouri et al. (2017) reported a significant decrease in ALT levels from 47.3 ± 5.4 U/L to 36.4 ± 6.6 U/L (P = 0.015) in pediatric NAFLD patients [11]. Similarly, Chahkandi et al. (2023) observed a reduction from 58.72 ± 32.16 U/L to 42.2 ± 20.2 U/L (p = 0.003), and Hashemi et al. (2009) documented a decrease from 113.03 IU/mL to 73.14 IU/mL (P = 0.001) in NASH patients, with ALT normalization achieved in 52% of the silymarin group compared to 18% in the placebo group (P = 0.001) [8,9]. The stratified analysis on the basis of etiology in this study demonstrated silymarin's greater effectiveness in HBV, HCV, and NAFLD subgroups, with ALT normalization significantly higher in HBV (23.1% vs. 10.0%, p = 0.038) and lower ALT levels in HCV (67.58 ± 22.41 U/L vs. 80.91 ± 23.64 U/L, p = 0.014) and NAFLD (42.43 ± 16.90 U/L vs. 67.92 ± 30.47 U/L, p = 0.013).

Similarly, AST levels improved in the silymarin group, from 86.52 ± 21.31 U/L at baseline to 57.08 ± 20.94 U/L after three months (p = 0.022), compared to a reduction from 89.88 ± 23.10 U/L to 65.90 ± 24.18 U/L in the standard group. Stratified analysis showed greater efficacy of silymarin in HBV and NAFLD-related CLD (p < 0.05). Chahkandi et al. (2023) reported a reduction from 36.62 ± 13.46 U/L to 30.3 ± 9.7 U/L (p = 0.036) in chronic liver disease patients regardless of the etiologies [8]. Additionally, Hashemi et al. (2009) observed AST normalization in 62% of silymarin-treated NASH patients compared to 20% in the placebo group (P = 0.0001) [9]. Similar results were reported by some of the previous RCTs [5,13,14]. The results of this study were consistent with the meta-analysis by Li et al. (2023), which confirmed significant improvement in ALT and AST levels across 26 RCTs [7].

The current study showed minimal improvement in bilirubin levels and coagulation parameters over three months, with similar outcomes between the standard and silymarin groups. Albumin levels increased slightly from 3.59 ± 0.47 g/dL to 3.68 ± 0.43 g/dL in the standard group and from 3.57 ± 0.46 g/dL to 3.69 ± 0.41 g/dL in the silymarin group (p = 0.940). Similarly, no substantial improvement was seen in INR and PT reduction (p = 0.746, 0.774). In the standard group, 48.5% were in Class A at baseline, which increased 64.7% after three months, compared to the silymarin group which increased from 54.8% to 67.1%, indicating comparable improvements at three months (p = 0.762).

Liver stiffness measured by FibroScan (EchoSens, France) decreased from 11.32 ± 1.20 kPa to 9.58 ± 1.13 kPa in the standard group and from 11.04 ± 1.13 kPa to 9.33 ± 1.04 kPa in the silymarin group after three months (p = 0.170). There was no significant improvement based on stratified analysis based on etiology at three months (p > 0.05). However, previous studies, including Wah Kheong et al. (2017) and Li et al. (2023), reported liver stiffness reduction compared to placebo (24.2% vs. 2.3%, p = 0.002) and (SMD = -6.64; 95% CI = -10.59 to -2.69), respectively [7,10]. Navarro et al. (2019), which reported fibrosis stage improvement in 12% (420 mg), 26% (700 mg), and 28% (placebo), suggest variability in silymarin’s impact across different dosages and patient populations [5].

Silymarin reported a greater reduction in BMI compared to the standard group after three months (p = 0.012). Hepatic encephalopathy absence improved slightly in both groups, from 82.4% and 83.6% at baseline to 89.7% and 87.7%, respectively, with minimal change in Grade 1-2 rates (p = 0.703). For ascites, the absence increased similarly in both groups (57.4% to 67.6% in the standard group, 58.9% to 67.1% in the silymarin group, p = 0.845). Parés et al. (1998) observed greater reductions in ascites (36% to 25%) and encephalopathy (13% to 8%) in the silymarin group, while the placebo group showed worsened encephalopathy (14% to 16%) [15]. While silymarin's impact on BMI was more pronounced in metabolic conditions like NAFLD, as demonstrated by Famouri et al. (2017) and Anushiravani et al. (2019), its effect on ascites and encephalopathy appears more limited in advanced cirrhotic states [14,16].

This study had several limitations. It was not conducted as a randomized controlled trial (RCT) due to financial, institutional, and patient-related constraints, making a non-RCT design more feasible for maximizing data collection and study applicability. Additionally, we did not evaluate silymarin's effects on other parameters, such as lipid profiles, quality of life, and in patients with decompensated liver disease. However, this study enrolled an adequate sample size compared to previous studies and included patients with chronic liver disease (CLD) irrespective of etiology, addressing a gap in the existing literature, which predominantly focused on NAFLD. A stratified analysis based on etiology provided more detailed insights for better understanding. Future studies should consider a larger multicenter RCT and a more diverse patient population to explore silymarin's effects across various etiologies and its potential role in managing decompensated liver disease.

## Conclusions

Silymarin demonstrated significant reductions in ALT and AST levels, with higher ALT normalization, particularly in HBV and NAFLD, compared to standard treatment. Hepatic encephalopathy and ascites improved similarly in both groups, and transitions to CTP Class A were comparable. Stratified analyses highlighted silymarin's greater impact on metabolic etiologies like NAFLD. Minimal effects were observed on bilirubin, albumin, and coagulation parameters.
